# Indents

**Published:** 1906-09-15

**Authors:** 


### INDENTS.

☞ **Brief Articles of Technical or Scientific Value will receive notice and comment. Contributions invited.**

**Air in Fillings.** The rotary burnisher is something different, and I do not remember that I have tried that and examined the results of the work. The result may be different from that obtained with the old burnishers. I was not surprised that Dr. Callahan found these little openings. I am surprised that he did not show more of them. And let me tell you that he might have shown just as many in some of our gold fillings that we are so proud of. I have seen gold fillings that were as bad and worse than the pictures he showed. I wish to compliment the doctor particularly on these illustrations. They are excellent. They look like the thing itself. I may surprise some of you when I say that most of your amalgam fillings are from 12 to 16 per cent air. The same is true of your gold fillings, but with amalgam it is almost impossible to get the air out.—G. V. Black *in Review.*

**Density of Tin-gold Fillings.** As regards the density of tin and gold. We find cells in all fillings, except these, as can be seen in specimens that I have with me.

As to the wearing out of the filling on the grinding surface of molars where from one-half to two thirds of the surface is exposed to wear and stress. The ten parts of gold to one of tin will stand that wear.

What Dr. Dittmar said of the filling being permeable to instruments is true, but that is the salvation of the filling. Being soft it spreads perfectly to the cavity, then after having been in use there comes a molecular change in the mass; and after a time, it will take a sharp bur to make an impression on the filling. This is a condition so generally recognized in tin-gold fillings that it was not mentioned in the paper.—J. R. Callahan, *in Review.*

**Why Use Tin-gold as a Filling?**

A very pertinent question, and one that is answered vaguely, 'tis true—in the paper—the cohesive gold foil filling is conceded to be the standard, but in the cavities mentioned tin and gold, as described, will last as well and can be put in, in one-third of the time that a cohesive foil filling can be introduced, thereby increasing the earning capacity of the dentist 33 per cent at least, for I claim the fee should be the same for one as the other.

I know it throws the average dentist into a fit—to try to convince him that he should get out of the day labor class, but let us hope that the day may come when the dentist will be able not only to make the dental supply people rich, but have a little left for himself.

As a rule, I do not approve of submarine fillings, but when they are necessary, tin and gold is the best material for filling such teeth. I do not mean a cavity that is full of saliva, but it is impossible to keep children from putting the tongue into a cavity during its preparation, thus moistening it. Before any one attempts to do this work, it is advisable to practice on extracted teeth to get the experience.—J. R. Callahan *in Review.*

**A Soldered Gold Crown from One Piece of Metal.**

After securing the root-measurement I cut the gold to the required length, so that when it is turned over and soldered, the band will exactly fit the root. Then I take a pair of shears and on one end of the band cut about twenty slits. Then I force these little sections over the end of the form which I have made to resemble somewhat the end of the tooth that is to be crowned. Take this band, which now roughly resembles a crown, place it on the root in the mouth, and trim approximately to the right length so that when the patient closes the mouth the occluding tooth will force itself into the soft end constituted by the sections lying one against the other. This gives a perfect articulation in most cases, and, as the slits readily yield, they can be bent until a perfect articulation is secured. The flux is then put on the outside of the articulating end of the crown and the solder on the inside; the crown is held over the flame so that when the solder melts a good backing of solder is obtained, which will permit of the crown being ground to suit the articulation. I claim for this method simply that we can secure a good fit of the root with the band, because we can see what we are doing. I also claim that by allowing the patient to force the teeth into this soft end we get a better occlusion than when the crown is made in the ordinary way. This is simply a little novelty that has proved quite helpfnl to me. —H. M. Schooley *in August Cosmos.*

**Impression and Dies for Root Cap for Crowns.** In order to make a metal cap or matrix, necessary in building a porcelain crown for a root, it is usual to take an impression in gutta-percha and from the gutta-percha impression, which is necessary more or less imperfect, make a die of Richmond or other fusible metal. The die when made is inaccurate and and clumsy. The whole method is slow and unsatisfactory.

After the top of the root is prepared, take a small stick of wood, pine preferred, about one-half inch square, whittle this down until it approximately fits the root to be crowned and slides easily between the abutting teeth, if there are any. Then drop sealing wax upon the pine stick, let the wax cool until it will no longer burn but is still soft enough to take an impression. Press firmly to place and hold till cold. You then have an excellent impression of the root of the tooth in intaglio, but, of course, in addition to that objection, a pine block does not make a good anvil. Now take your impression and oil it, then take the anvil block of your rubber swager, melt the sealing wax upon it and press the pine block quickly and firmly to place, using no twisting or sliding motion. After cooling, these two sealing wax surfaces will separate leaving the perfect die wanted and upon an anvil ready for the swager. These operations can be completed in much less time than I have taken to tell it and are more accurate than any other. The metal cap may then be returned to the tooth for finishing.—Dr. C. C. Allen *in Brief.*

**Gold Filling with Cement Protection.** My present method is to take the oridnary cement—colored to match the tooth by using various colors—in a very small quantity on the point of a small probe, working it all over the cavity. Take porous gold, ''Watt's Crystal,'' ''DeTrey's,'' or ''Moss Fiber,'' and press it into the cement, covering the entire cavity, and with small hot burnishers in each hand hold the gold from rocking or getting soiled with the cement on the outer surface. We pack with smooth, large neck burnishers the greater part of the filling, burnishing the gold eachtime with *hot instruments,* when the next piece will adhere readily. Instruments must be heated about as hot as can be, without drawing the temper—using them while hot. The wet cement under the gold will prevent thermal changes. When the filling is two-thirds made, take a paring chisel and bevel the enamel edge, then place the next piece of gold at the center of the filling and burnish back onto the enamel until the filling is complete.

Having practiced these methods with some advanced improvements each year for about ten years, and watched with great care the comparison with other and older methods, many have come to fully appreciate the advantage in preserving tooth structure, and believe it is soon to be the popular and most practical method of filling teeth. We believe gold and amalgam are unfit materials to use in contact with the teeth, but should be used as a secondary matter to protect the cement from the fluids of the mouth, while the cement is the real and only practical preserver of teeth from the ravages of decay.—Dr. L. C. Taylor *in Brief.*

**Guarding the Cavo-incisal Edges of Inlays with Gold.** Such cavities in the incisors are usually prepared with the cavity margins at an angle of ninty degrees to the incisal edge, so that the joint will be parallel to the long axis of the tooth. After such fillings have been inserted it has been my experience that the pressure of soft food at this edge will pump out the cement, and consequently we always find the cement washed out to a greater depth at this joint than at any other in the filling. In a short time these walls being unsupported, with both the tooth and porcelain edges exposed to the full blow of the bite, check, resulting in the final loss of a piece of enamel or porcelain, leaving an unsightly crack. When such an accident occurs, and they have occured in my practice in a short time after the operation-what shall we do? I have found nothing better to do, heretofore, than to remove the filling, and prepare another inlay, which means the destruction of more tooth-structure than any conservative man is willing to attempt.. My method now is to divide the incisal edge at the cavo-incisal angle, cutting back into the palatal wall. I make the filling in the usual way and then grindoff a bit of the porcelain extending into the step, leaving the cavity supported with sound dentin. The filling between the approximating teeth is supported by the aid of wedges, and with everything in readiness, the cavity is fllled with a thin lining of cement, and a small piece of thin DeTrey's gold is laid in the palato-incisal angle, allowing it to extend beyond the cavity. The filling is covered with cement and is pushed into position, the wedges being left untouched until the cement sets, when the gold is burnished off. In this way two layers of cement are utilized, one on the tooth and the other on the inlay, while the gold between them guards the edge in such a way as will prevent any pumping out of the cement from that joint. Furthermore, if the cement does pump out, the joint will not permit of daylight showing through it.

I have found it good practice to place in the palatal edge of inlays a piece of gold foil when cementing them to place.

This is put in while the cement is soft, and with the cement on the inlay in the usual way, it is wedged into position, giving a filling made on this principle with as little cement as possible in the joint.—C. J. Grieves *in August Cosmos.*

**Alypin.** In the June Register, page 305, was published an extract from the Dental Cosmos on "Alypin," in which we made an erroneous quotation. To correct the matter we herewith print the entire review by Dr. Endleman as it appears in the March *Cosmos*, at page 356. The article is a review of some experiments made by D. L. Camus and is printed in the January 1906 —L'Odontologie, *Paris, France.*

This newly isolated local anesthetic is being hailed by a number of continental practitioners as a succedaneum of cocain, stovain, and the other agents of the analgesic group. Weak solutions anesthetize the cornea; in two per cent. solutions it is innocuous when applied to the visual structures; it anesthetizes the mucous membranes when rubbed upon these surfaces and also the skin when hypodermically injected.

From the experiments made upon dogs, the author is enabled to state that in some cases the fatal dose does not exceed $\frac{1}{2}$ gram of alypin, per kilo of body weight, ($2\frac{3}{4}$ pounds) an amount about equal to the fatal dose of cocain which has been found to vary between 3 to 6 grs. It was found on the other hand, that a non-fatal dose of alypin of one and one-half grains caused in the animals experimented upon a series of toxic phenomena similar to the poisonous effects exhibited by cocain. A dose of five milligrams per kilo of body weight causes invariably a decrease in blood pressure; one of ten milligrams, besides further decreasing the blood pressure, inhibits cardiac activity and increases the amplitude of the pulsations, while a dose of twenty milligrams rapidly decreases blood pressure and soon causes death.

The cardio-vascular action of alypin should not be looked upon as a barrier against its use, inasmuch as the effect of five milligrams doses per kilo of body weight is neither marked nor persistent, the blood pressure becoming normal inside of five minutes. Furthermore, as has been pointed out by the originators of alypin, the cardio-vascular action can be greatly modified by the addition of a small amount of an adrenalin solution to the anesthetic mixture. The general toxicity of the agent should not constitute a source of any great anxiety, when it is considered that in the most unfavorable experiment of the series above referred to, to the symptons were those caused by cocain. The dose of five milligrams per kilo of body weight with which the author experimented, and which does not give rise to convulsions or to any other dangerous manifestations, would be equivalent in the case of an adult of average weight, to a dose of from 0.30 to 0.35 gram (gr. v to vj), or in other words, to from 15 to 17 cc. of a 2 per cent. solution.

In concluding the author adds that his experiments were performed by injecting the several doses of alypin directly into the circulation through the femoral vein, a method which is never employed in surgical practice, and one which induces quicker and more active results than the ordinarily employed hypodermic method.

**Immutability vs. Progress.** As an illustration of what would appear to be a complete somersault is Crouse in 1875 recording his solemn conviction that "if there had been no such thing known as amalgam humanity would have been better off!" And Crouse in 1906 manufacturing tons and tons of the most popular amalgam in the market! Now the history of the profession is filled with just such reversals, and I am not calling attention to them here as illustrations of inconsistencies, or of anything else that could in the least be construed into a reflection on their judgment or to point a warning even for greater caution. Quite the contrary. The point I want to make is just the reverse. These changes of attitude, instead of being a reflection, are a credit; instead of showing weakness, they indicate strength and activity, and are as necessary to growth as air is to our existence. Dr. Crouse himself met the question in exactly the right way when he was at one time twitted with advocating what he had previously denounced but a short time before. They thought they ''had'' him, and of course there was a laugh at his expense. Now, when you want to ''get'' Dr. Crouse I have observed it is quite necessary to get up early in the morning; in fact the day before wouldn't be any too soon. Yes, he replied, he knew he had advocated other modes of practice at previous meetings, and he regretted if he had done harm by so doing. Nevertheless, he always reserved the right to change his mind when he found he was wrong, and if during the coming year new light, new knowledge, should cause him to change his mind again, he wouldn't be afraid to admit it. Now, there spoke the honest seeker after truth, the student who was more concerned to be in the right than to try to impress others with his infallibility. This is the very essence of the spirit of progress. He writes himself down a stupid ass, who having once nailed his flag to the mast, obstinately defends it, in spite of changed conditions, just to be consistent. Without a mind wide open to conviction, progress is impossible.

And this is the point I have aimed to bring out for the especial benefit of those who have lately joined with us and who are on the threshold of their professional or society careers, I say to you, it is your duty to be in the thick of it and know what is going on. If a new theory or new method is suggested, it is your duty to see what there is in it. You don't want to wait for others to investigate for you. He who stands back and waits for others to find out first whether this methods or this practice is a good thing, isn't worth a ''hill of beans,'' either to himself or his profession. He is losing a lot of valuable time and thereby checks his growth, stunts himself, stays in a rut, becomes a back number, and finally dies of dry rot. You want to be a ''helper,'' not a ''learner,'' as Dr. Thorpe once poetically expressed it. If there is anything advanced that may enable you to do better work and serve your patients better, you want to ''get onto it'' at the very earliest moment. You can't afford to wait and let others get there first. You want to be in the van; you don't want to struggle along in the rear—and when you once have satisfied yourself that a method or theory is right, you want to advocate it and make others see the right. Suppose you will have to reverse yourself at times Suppose you do have to ''eat crow'' at times; that's all right. You will be in good company. Crouse and Black and the host of others who have made their marks in the profession, all have ''eaten crow'' in their time, and will continue to do so, it is safe to say, as long as good red blood flows in their veins, and they are good enough company for anybody. They haven't lost standing because of it. ''Chewing crow'' is good for the teeth and stomach. It will only strengthen your digestive apparatus and you will be all the better able to cope with and assimilate subsequent propositions as they come up. The mere effort expended in personaly investigations and experimenting is worth more educationally than the study of either books or lectures.—Dr. Rohland *in Review*.

**General Anesthetic.** Dr. Eli H. Long in the August *Cosmos*, discusses in a most practical way the principals underlying the selection of general anesthetics from which we select the following paragraphs.

Two dangers must be considered: First, that of depressing a heart that is already at a disadvantage, and second, that of lessening the oxygenation of the bood, which might lead to embarrassment of the heart by distention of its chambers; for venous blood does not pass readily through the capillaries. Nitrous oxid as usually given, without oxygen, would present the latter danger, and chloroform the former, because of its marked depressant action upon the heart.

The condition of the bloodvessels claim careful attention, for the important reason that they furnish a fairly reliable index of the progress of the dengerative changes in the system as a whole. The changes which they are most subject to are essentially those of age, whether they represent a normal senile degeneration late in life, or an earlier disease of the arteries due to such causes as syphilis and alcoholism. So definitely has this come to be recognized as to lead to the axiom that "a man is as old as his arteries," meaning that a man may be old at forty because his arteries show the changes that belong to advanced life. The general name arterio-sclerosis is given to the condition. The changes consist of a hardening, a weakening, and a compensatory thickening of the vessel walls; in consequence theyb ecome less elastic and less fit to stand the strain of the increased blood pressure that naturally accompanies the disease.

The diagnosis of arterio-sclerosis is based largely upon the palpable thickening of the radical and temporal arteries, with accentuation of the aortic second sound of the heart, which indicates high arterial blood pressure.

Two dangers must be noted as attaching to arterio-sclerosis in relation to general anesthesia: First, with the lessened oxygenation of the blood that occurs when the supply of air is limited in the administration of an anesthetic, the blood pressure is believed to increase in the areas of the smaller vessels—the arterioles—because a blood poor in oxygen and surcharged with carbon dioxid stimulates the circular muscle fibers of the arterioles to constraction; thus the blood is held back in smaller arteries instead of passing freely on into the capilaries. If the vessel walls be weakened by disease, the local increase in blood pressure may cause rupture, which, if occuring in the brain, would mean apoplexy. This danger has been recognized for some time as pertaining particularly to the use of nitrous oxid, because of the attending cyanosis, so that arterial disease is commonly held to be a contra-indication to nitrous oxid. Second, another danger, more obscure as to its recognition, is that of degeneration of the heart secondary to arterial disease. It may not be apparent by the usual means of physical diagnosis, for under ordinary influences the function of the heart may not be sensibly disturbed. The danger cases are precisely of the kind that terminate in sudden death in the midst of life's activity. Degeneration of tissues has begun, and we can think of such a case as one where death has laid its finger upon parts of the body that are not in themselves so virtually important, but which, by progressive changes, originate secondary influences that affect the most vital structures of the nervous system and heart.

In the case in which any question of safety arises, our judgment must be concerned about the choice of an agent no less than about the condition of the subject, for it does not follow that if our judgment interdicts one agent it must prohibit all. Here we must apply the rule that the drug that is least liable to disturb circulatory conditions should be given first place, providing it does not unduly increase the danger of toxemia, either directly by its own action or by embarrassing elimination.

The objection to nitrous oxid that it induces asphyxia, can be overcome by combining sufficient oxygen with it to maintain a good degree of oxygenation of blood, with which modification it is unquestionably the safest of all agents wherever it is applicable.

The chief objection to ether that is held to be a real danger—that of the large amount needed, which may seriously tax the eliminative organs—may be largely removed by preceding its use by either ethyl chlorid or nitrous oxid. The fact that it is not a heart poison gives it a place of permanence as the safest known agent for a profound, continued effect. It induces practically no disturbance of circulatory conditions.

The objections to chloroform have reference to its circulatory effects chiefly. While experimental evidence show that chloroform kills the healthy mammal by paralyzing respiration—and cases are seen where, respiratory failure occurring under this drug, the patient is promptly resuscitated by artificial respiration—yet its great danger lies in the fact that it greatly depresses the heart, and its free or prolonged administration may cause fatty degeneration of that organ. The irritability of the heart muscles is also reduced, so that its response may be deficient. With chloroform, therefore, when respiration stops we have to reckon not only with a paralyzed respiration, but with a depressed heart as well. The odds may be too great for the organ to recover itself, hence the frequent fatalities from this anesthetic.

